# The Use of Acoustic Emission and Neural Network in the Study of Phase Transformation below M_S_

**DOI:** 10.3390/ma14030551

**Published:** 2021-01-24

**Authors:** Małgorzata Łazarska, Tadeusz Z. Wozniak, Zbigniew Ranachowski, Andrzej Trafarski, Szymon Marciniak

**Affiliations:** 1 Institute of Fundamental Technological Research, Polish Academy of Sciences, Pawińskiego 5B, 02-106 Warszawa, Poland; zranach@ippt.pan.pl; 2 Institute of Materials Engineering, Kazimierz Wielki University, Chodkiewicza 30, 85-064 Bydgoszcz, Poland; wozniak@ukw.edu.pl (T.Z.W.); trafarski@ukw.edu.pl (A.T.); 3 Faculty of Materials Science and Engineering, Warsaw University of Technology, Wołoska 141, 02-507 Warszawa, Poland; szymon.marciniak@nanostal.eu

**Keywords:** bainite, austempering, acoustic emission, neural networks, dilatometry

## Abstract

Acoustic emission and dilatometry were applied to investigate the characteristics of phase transformations in bearing steel 100CrMnSi6-4 during austempering below the martensite start temperature (M_S_ 175 °C) at 150 °C. The aim of this study is to characterize the product of transformation occurring below the M_S_ temperature using various research methods. Analysis of the dilatometric curves shows that, after the formation of athermal martensite below the M_S_ temperature, the austenite continues to undergo isothermal transformation, indicating the formation of bainite. Additionally, tests were carried out with the use of acoustic emission during isothermal hardening of the adopted steel. The obtained acoustic emission signals were analyzed using an artificial neural network. The results, in the form of a graph of the frequency of acoustic emission (AE) event occurrence as a function of time, make it possible to infer about the bainite isothermal transformation. The results of this research may be used in the future to design optimal heat treatment methods and, consequently, may enable desired microstructure shaping.

## 1. Introduction

Great technological progress in recent times has caused demands concerning used materials to continue increasing. Most of these materials are metals. Contemporary materials science, materials engineering, and structural metallurgy tend towards the manufacture of materials that meet the requirements of more and more challenging tasks of different industrial sectors. One of the methods that may be used to considerably improve the quality of metals is heat treatment. The first research on the effects occurring during austenite decomposition at a constant temperature was done for eutectoid steel by E. C. Bain and E. S. Davenport in 1930 [1]. The results of these studies led to many other investigations on the isothermal decomposition of austenite. Bainitic transformation is still a not fully investigated phase transformation from all transformations of austenite in steels. The kinetics of bainitic transformation is controlled by the nucleation rate [2,3]. Bainite is a structural component that is interesting for many phase transformation researchers. The mechanism of bainitic transformation is still not understood completely and remains a subject of research since its discovery.

Isothermal phase transformation below M_S_ was examined by many researchers [4,5,6,7,8,9,10]; however, its nature has also still not been fully explained. Most of the research was done on hypoeutectoid, a highly alloyed steel with a significant content of Mn. The kinetics of transformation in steels with contents of Fe-0.85 wt%, 10 °C below M_S_, was also studied by H. Okamoto and M. Oka [11,12]. The isothermal product formed below temperature M_S_ differed from traditional lower bainites, which are formed above M_S_ temperature. However, after detailed studies, it was concluded that, in austempered carbon steels, the formation of bainite was preceded by a formation of midribs. The effects of acceleration in this respect were explained by the influence of midribs on bainitic transformation. Since 1955, O. Schaber [13] was the first to notice the occurrence of two-stage transformation near the M_S_ temperature. He observed while examining steels 0.36 ÷ 1.08%C that the transformations overlapped near M_S_. The author supposed that martensite was formed at the first stage and that it preceded to form isothermal bainite. In the low temperature area, the changes in the kinetics of bainitic transformation provoked the kinetic curves in the TTT (Time Temperature Transformation) diagram to form the shape of the letter S. Although it was confirmed many times in the literature, the mechanism of transformation has not yet been recognized. S.M.C. van Bohemen et al. [5] confirmed that bainite can be formed below M_S_ for steel 0.69-Mn, 0.66-C wt% within the temperature range 220–300 °C. Based on dilatometric tests and scanning microscopy, it is concluded that, after the formation of martensite below M_S_ 264 °C for this steel, austenite is still transformed isothermally into a product of more bainitic than martensitic character. P. Kolmskog et al. [7] is of the opinion that bainitic ferrite may be generated at a temperature near the temperature of martensite formation. Bainitic ferrite grows in the same way as Widmanstätten ferrite above M_S_. The research done by Kolmskog aimed to confirm the presence of martensite and bainite at the same temperature. S. Samanta et al. [8] identified the product of isothermal transformation below M_S_ to be bainite. He pointed out that the kinetics of transformation is in accordance with a model based on the formation of bainite by a displacive mechanism. However, a microstructural analysis showed that bainite and martensite have similar crystallographic characteristics. Elisabete Pinto da Silvaa et al. [6] also claimed, as the result of various combinations of testing methods for steel 0.16-C, 1.6-Mn wt%, that the product formed below M_S_ temperature is mainly of bainitic character.

Acoustic emission (AE) is a testing method that is now widely used in various fields of science for different purposes. It is also used in the study of metals, enabling the testing of effects while austempering. Testing the acoustic signals is currently being carried out at many laboratories [14,15,16,17]. The aim of these tests is to determine the most characteristic features of the signal reflecting the transformations occurring in the material and the defects arising during manufacturing. AE measurement automation also enables independent control of the selected technological processes. Recording a full course of signals allows for widening of the measurement range and the use of frequency characteristics. The detection of defects and faults enables the user to make a correct diagnosis of building structures. Acoustic effects arise from sudden liberations of cumulative energies in materials [18,19]. The sources of acoustic waves in steels are phase transformation, dislocations, macro- and micro-cracks, and radiation [5]. A very precise and sensitive research method was used to control these occurring phenomena. The results obtained indicate that the applied methodology is a very effective and precise method in this type of research.

An analysis of the signals received from steel hardening provides information about microstructural changes in the processed material, such as microcracks arising while hardening, during dislocation processes, and especially during phase transformations [14]. The aim of this analysis is to determine the most characteristic features of a signal depicting the history of change in a material. The possibility of following the kinetics of phase transformations during hardening processes may facilitate the development of new heat treatment methods. Improved methods of processing significantly affect the quality of the products. Artificial neural networks (ANNs) were used to analyze the AE signals.

ANNs are widely used to address specific tasks such as information processing, identifying speech and objects. They are applied in the diagnostics as well. They enable the analysis of several processes and the condition of materials used to manufacture many elements. Recently, a growing interest in artificial neural networks has been observed. These networks are also used in the study of metals as a research tool to analyze events present while austempering steels [15], which is shown in this study. There are also other new and interesting research methods known in the literature that can be applied to the study of phase changes. In particular, it is worth mentioning thermodynamic simulation [20] (aimed at achieving holistic management of the phase assemblages of alkali-activated materials (AAMs) and gaining insights into the designing of precursors). Another method to analyze changes could be molecular dynamics (MD) calculations based on the potentials of interatomic interactions [21].

The aim of this study was to analyze austempering below M_S_ using various research methods. The application of AE to investigate phase transformations in bearing steel is presented here. In addition, a dilatometric research (DIL) was made to demonstrate the possibility of bainite formation below M_S_ temperature.

The dynamic development of technology forces the search for new, more efficient, and safe technologies. Proper selection of the heat treatment method has a fundamental impact on the optimal use of ready-made elements. The current market forces accurate analysis and control of the state of materials, which are elements in the design and implementation of equipment. Knowledge of the phenomena occurring during heat treatment is the basic condition for the production of products with appropriate functional properties. Therefore, research methods are being sought to enable tracking of the phase transformations in steels during hardening. Nowadays, various methods of testing nondestructive materials are known. Among them, the acoustic emission method plays a very important role. The use of this test method makes it possible not only to track cracking and damage processes but also to study the phenomena occurring during steel hardening. Many years of research using acoustic methods in the field of bainitic transformation make it possible to use them in the industry. In addition, they can be used in the design of optimal heat treatment methods and in the shaping of the microstructure.

## 2. Materials and Methods

Bearing steel was used to perform the tests. An analysis of the chemical composition of the tested materials was performed on spark spectrometer BRUKER model TASMAN Q4. The results are shown in Table 1.

**Figure 1:**
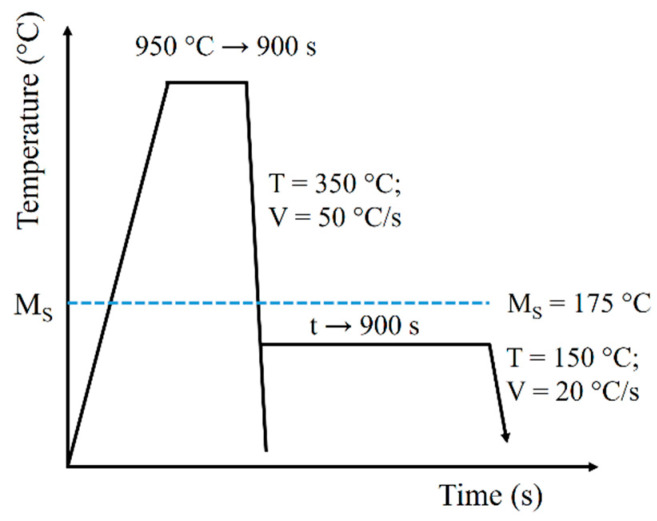
Schematic diagram of thermal treatment with marked recording time limits and dilatometric measurements.

Dilatometric analyses were carried out using a hardening dilatometer DIL 805 L with a quartz measuring system, induction heating, and cooling achieved by blowing with compressed helium of purity 99.999%. Specimens of a diameter of 2 mm and a length of 10 mm were prepared to perform the dilatometric tests. To examine transformations below M_S_ temperature, the specimens were austenitized at 950 °C for 900 s. Then, they were cooled at a rate 50 °C/s to a temperature of 350 °C. After that, they were cooled at a rate of 20 °C/s to a temperature of 150 °C and were held at this temperature for 900 s. Then, the specimens were cooled to room temperature. A schematic diagram of the process is illustrated in Figure 1.

**Figure 2:**
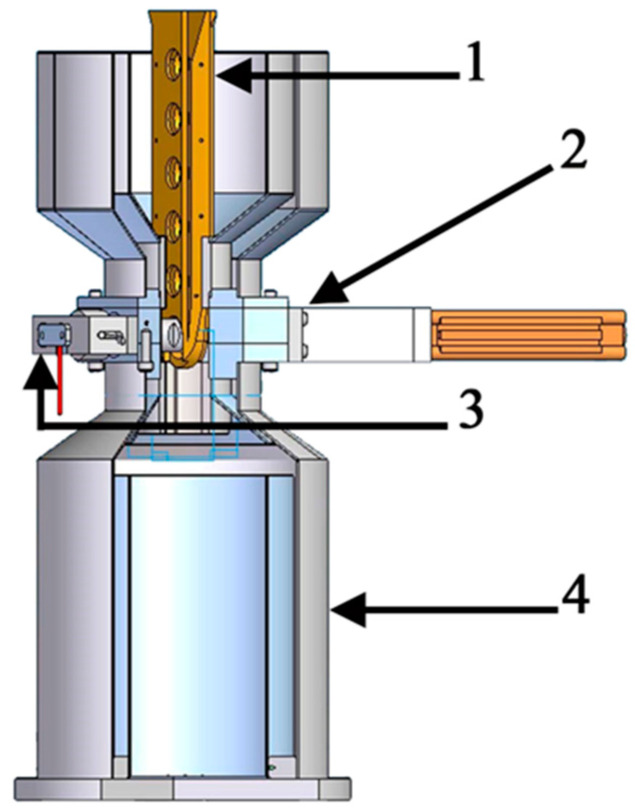
Research workstation for the acoustic emission (AE) tests: 1—guide, 2—ultrasonic sensor, 3—waveguides, and 4—tank.

AE tests were conducted in a special workstation designed for this purpose, which made precise measurements possible. The workstation was equipped with an instrumentation and computer hardware with specialist software. A diagram of the research workstation designed in the Solid Edge environment is shown in Figure 2.

An important element of the workstation is the control system equipped with a relay to control the pneumatic actuator used to clamp specimens during the test and the pressure indicators on both the actuator and the regulator. The two regulators of oil temperature in the tank are significant parts of the system. A runner installed inside the oil tank is used to transport tested specimens. The reception of AE signals was made using an ultrasonic sensor.

**Figure 3:**
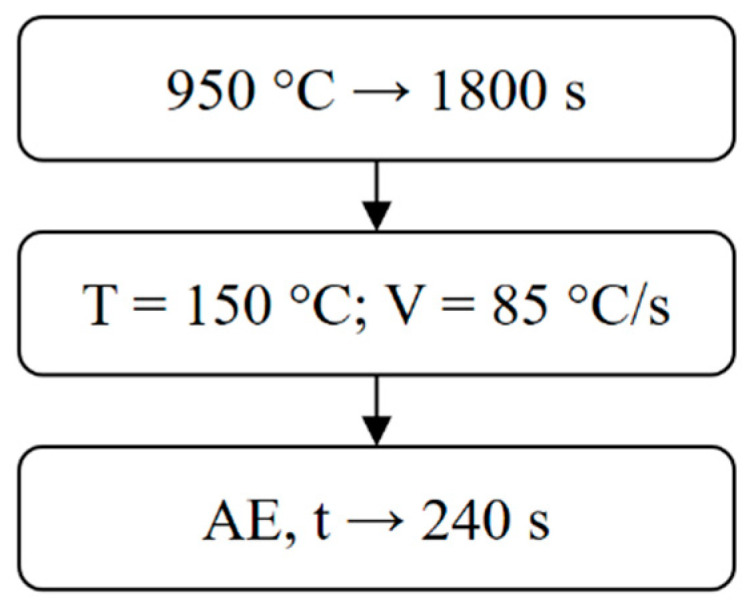
Flow diagram of the thermal treatment with marked recording time limits and acoustic emission (AE).

Specimens of diameters of 45 mm and 2 mm were austenitized at 950 °C for 1800 s. Then, they were put in the runner installed in the tank filled with oil heated to a temperature of 150 °C. This allowed the transport of the tested specimen to the area of contact with two mobile waveguides. In the next step, the waveguides were clamped to the specimen. The ultrasonic sensor was installed to the one of waveguides. In the last step of the test, the AE signals were registered in the PC memory. The process of thermal treatment is presented in Figure 3 in the form of a flow diagram.

**Figure 4:**
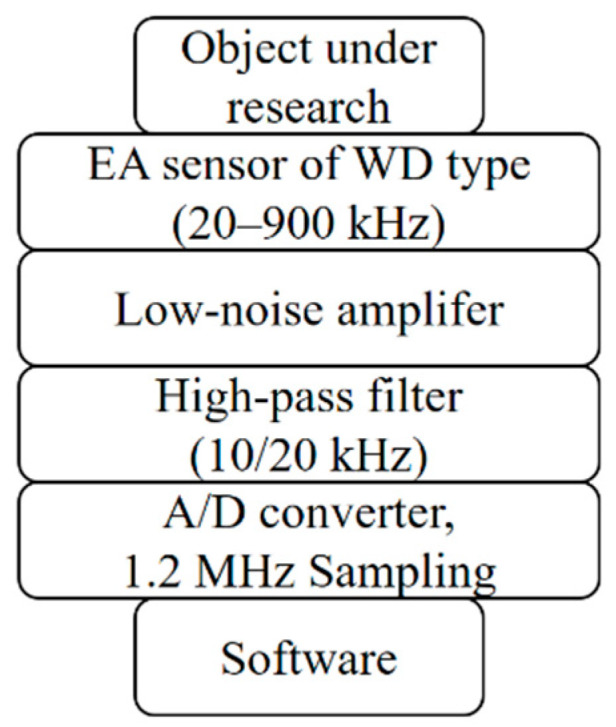
Block diagram of the specialist workstation for testing AE signals.

The AE signal in the form of elastic waves was converted into an electric signal using piezoelectric transducers. Then, the signal was sent to the analyzer, where its initial processing was made and the result was stored in PC memory. Recording was performed using Digital Adlink-9812 on an analogue-digital card. A diagram of the workstation for testing AE signals is shown in Figure 4.

The specialist workstation for testing AE signals was equipped with an AE analyzer with a sampling of 1.2 MHz. A high-pass filter with a cutoff frequency of 20 kHz was applied. This eliminated background signals from further signal processing. Moreover, a low-pass filter was also applied, the aim of which was to limit noise and to make the signal smoother. The results were presented in a form of spectrograms. The Short Time Fourier Transform (STFT) algorithm with Hamming window was used to obtain these spectrograms. The presented measurement system made it possible to record the EA signal in 240 s. After completing the registration on the hard disk of the control computer, a file in .wav format was generated, the size of which was 576 Megabytes. The whole procedure enabled the comparison of the results obtained in the form of signals for the selected frequency intervals. The spectrograms were obtained by dividing the AE signal into smaller fragments and by calculating the components.

In the received signal of acoustic emission sounds, a large number of AE events was registered. In this article, an artificial neural network was used to filter the signal of acoustic emission in order to more accurately determine the beginning and end of the phase transformation. For this purpose, software consisting of three programs was used. The first of them divided the registered signal into segments of durations of 7.5 ms. In each segment, the AE events were detected and their average energy was determined. AE events that differed from one another by spectral characteristics and energy were identified. Before and after the transformation, there were signals with energies lower than the adopted level for signals from the phase transformation. Strong and weak AE events were also recorded during the phase transformation. Energy intervals were determined by dividing them into the events of high (above 10,000 pJ), medium (1000–10,000 pJ), and low (below 1000 pJ) energy levels. The data were selected as a result of the analysis of the signal divided into segments taking into account the energy of events and their spectral characteristics. There were generated spectral characteristics of these AE events with predetermined energies. When considering the continuous acoustic emission signal in a selected time period, we can present it in the form of the sum of its frequency components. This means characterizing the signal not in the time domain but in the frequency domain. Assuming absolute integrability of the function, it can be linearly transformed into a spectral density function using the Fourier transform. In the case of a discrete signal, the set of its samples was divided into segments corresponding to time intervals convenient for analysis (i.e., 18,000 samples each). In these intervals, the discrete form of the spectral density function was determined. The algorithm transforming the set of signal samples into a set of spectral density coefficients was implemented according to the following approximate formula:

(1) $$c_{n}\approx \frac{1}{N}\overset{N-1}{\underset{m\;=\;0}{\sum }}v\left(m\cdot T_{1}\right)\cdot \mathrm{mod}(e^{-\frac{jn2\pi m}{N}})$$

where *j* means $\sqrt{-1}$ and mod means the absolute value of the complex expression

The feature vector (sequence of zeros and ones) is sent to the input layer and then propagated to the output layer through interneurons. The frequency–energy relationship was introduced into the neural network. These were vectors of 25 units describing successive frequency ranges; there were 80 vectors. The spectral characteristics on the horizontal axis shows the frequency range from 0 to 600 kHz, and the vertical axis shows the interval energy density of the measured signal. The software calculated this energy density in frequency increments of 7.5 kHz. There were 80 of these intervals. Energy density was measured in decibel units, that is, on a logarithmic scale. The zero decibel level was adopted for the signal for which RMS voltage during the measurement was 1 millivolt. The unit for neural processing of the pattern energy level was 2 decibels. The values of the coefficients were processed using a logarithmic function (similar to audiology) in such a way that the result values were in the range of 0–10. Signals in the range from zero to 1 can occur on outputs of the neural network depending on the pattern given on the input and on the values of the weighting factors that were saved during training of the network.

**Figure 5:**
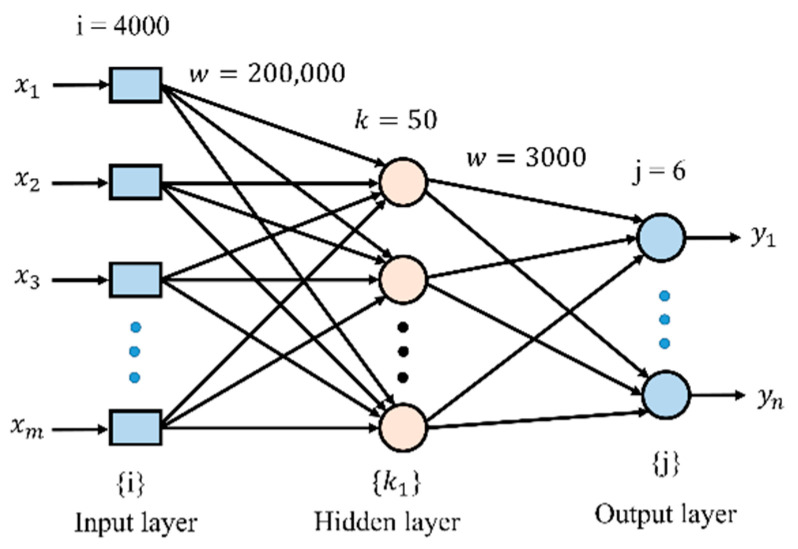
A simplified diagram of the neural network used in the research.

The neural network was made of neurons arranged in two layers. The first table of weight coefficients contains 200,000 coefficients imitating links between the units of the input layer and the 50 units of the next layer. The other table included 3000 coefficients imitating links between the units of the second layer and the six units of the output layer. A simplified diagram of the neural network used in the research is shown in Figure 5.

A signal was generated at the network outputs depending on the degree of similarity of the input vector and the remembered patterns (feature vectors).

The following patterns and network learning sequence (learning scheme) were used:

- A1 (14,058 pJ) and A2 (14,970 pJ), high power AE signal patterns,
- B1 (5411 pJ) and B2 (3128 pJ), medium power AE signal patterns,
- C1 (53 pJ) and C2 (60 pJ), background patterns.

Learning scheme for 3 patterns: (16 × A, 8 × B, 4 × C).

The learning sequence was repeated 6 times in an alternating cycle.

A feature of the neural network is the ability to autonomously modify its organization, i.e., change the matrix of W. This process is called “network learning”. The “back propagation” was used to train the artificial neural network. The activation function in the form of a sigmoid was used. During the network learning procedure, according to the “back propagation”, an experimentally selected sequence of patterns was given to the network inputs and the state of the network outputs was forced in such a way that one of the network outputs had the maximum signal level when a specific pattern was given to the input. Thus, there was a signal at the network outputs that could be used to classify the vectors given to its input. Illustratively, the relationship between the input vector X for each network layer and its output vector Y can be presented as follows:

Y = W*X

where W is a matrix containing a weight matrix and * means the operation of mapping the inputs of the network layer to its outputs.

The W coefficient matrices are in fact tables of coefficients describing the weights of the connections between successive layers. In the process of memorizing the given patterns, the values of the weight tables are modified in such a way that, when the data vector is given to the input layer, the output receives a vector which is a measure of the similarity of the tested data set to the previously established reference vector stored in the weight tables. The process of memorizing (one or several) patterns takes place automatically using an iterative algorithm. When considering the feature vectors processed in the network as strings of zeros and ones, the relationship between the signal at the input yj of a single neuron and the signal at its output yi can be written as follows:

yi (t + 1) = θ (Σj wij yj (t) − µi)

where θ is an activation function, most often in the form of a sigmoid 1/(1 + exp(−x)); x is the net input the respective processing elements; wij is called a weighting coefficient with a synaptic weight which expresses the bonding strength between connected neurons labelled j and I; and µi is the process parameter, called the threshold level.

After completing the learning procedure, the neural network gained the functionality of classifying sets of spectral characteristics provided at its input in terms of their similarity to the learned patterns: A, B, and C.

The program enabled carrying out the network learning procedure, tracking the state of the network during the learning procedure, and testing the state of the network outputs after providing spectral patterns of AE events to its input. The neural network method was also used to analyze the AE signal in the authors’ work [15,22].

**Figure 6:**
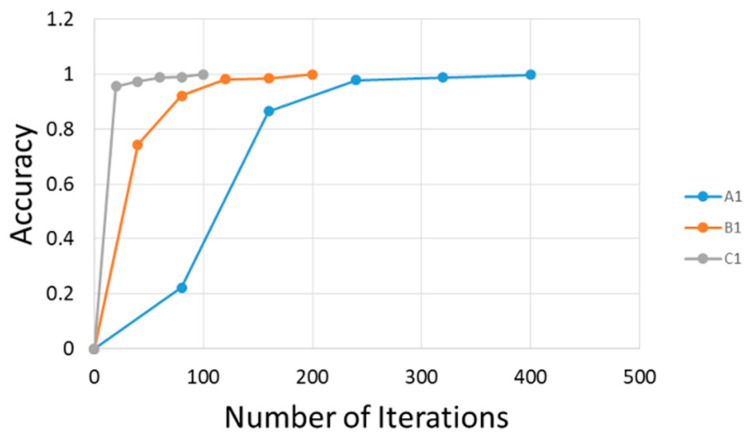
The accuracy of the initial weight table.

After correctly carrying out the process of the learning network, it was possible to investigate the responses of the network outputs to the adopted three patterns. For patterns A1, B1, and C1, 504 epochs were used. The accuracy the training of the artificial neural network is shown in Figure 6.

## 3. Results

**Figure 7:**
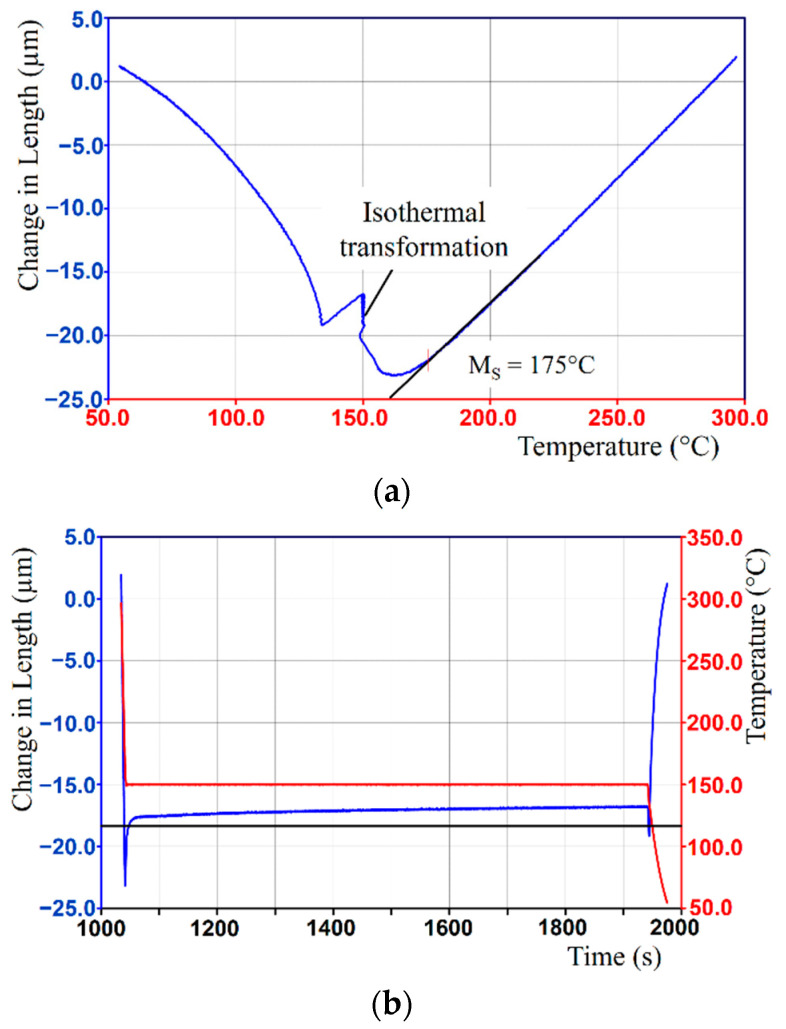
Results for dilatometric tests for bearing steel under isothermal conditions below M_S_ temperature: (**a**) details of the isothermal transformation and (**b**) an enlarged view of the region of complete thermal cycle.

The M_S_ temperature determined on the basis of the dilatometric tests was 175 °C. Dilatometric tests were made to investigate the transformations of austenite under isothermal conditions below the determined M_S_ temperature. The test results are shown in Figure 7.

As was observed from the dilatometric curves, the isothermal transformation associated with the increase in sample volume starts at the time of holding the specimen at a constant temperature of 150 °C. In the thermal conditions adopted, an effect of dilatometry changes on the tested specimen. At that time, the formation of athermal martensite in the alloy was interrupted and, then, a bainitic transformation occurred. The dilatometric tests were carried out with a change in cooling rate to eliminate the impact of the factors that induce the acceleration of bainitic transformation. The adopted thermal conditions enabled austenite homogenization and stress decay.

**Figure 8:**
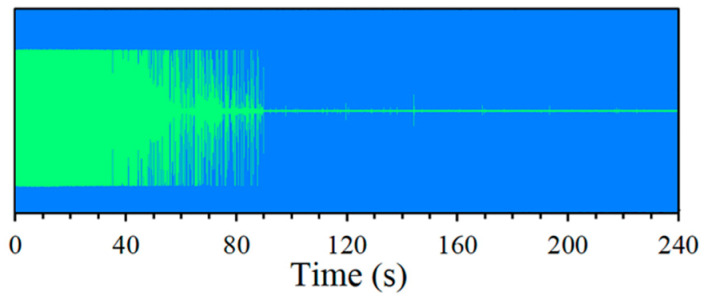
Record of the complete AE signal for bearing steel from isothermal quenching at a temperature of 150 °C.

To confirm the dilatometric results indicating the occurrence of bainitic transformation below M_S_ temperature, additional tests were performed using highly sensitive testing techniques, i.e., acoustic emission. Artificial neural networks were used to analyze the received signals. Isothermal quenching was made with the use of specially prepared specimens of bearing steel at 150 °C according to the flow diagram given above. The results on a form of AE signals are presented in Figure 8 below.

**Figure 9:**
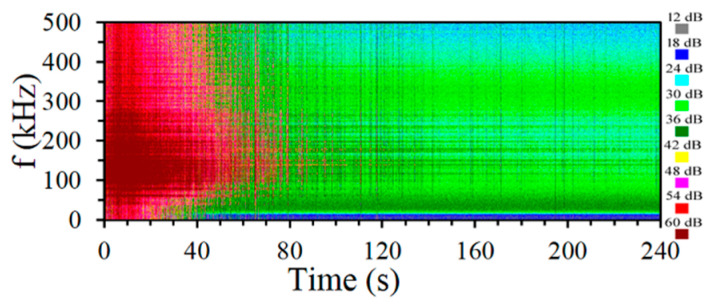
AE signal spectrogram for steel bearing during austempering at 150 °C: the colors encode the square root value of the signal power spectral density.

As was observed from the results in a graphic form, the start of the signal shows many explosions of AE events. A significant intensity of signals occurs during the first 100 s of recording. The intensity of signal generation for the tested steel indicates the occurrence of phase transformations. A spectrogram of AE signals was created for bearing steel and is shown in Figure 9.

AE signal spectrograms made for bearing steel while austempering at 150 °C present square root values that define the power spectral density in a 9-stage scale with a 6 dB step. The lowest value is marked in grey, and the highest one is marked in brown. The spectrogram presented is a graph of the spectrum recorded during testing. It can be the basis to conduct the analysis for a pick-and-mix frequency. Components of the spectrum obtained using Fourier analysis indicate the highest intensity within 100–300 kHz.

**Figure 10:**
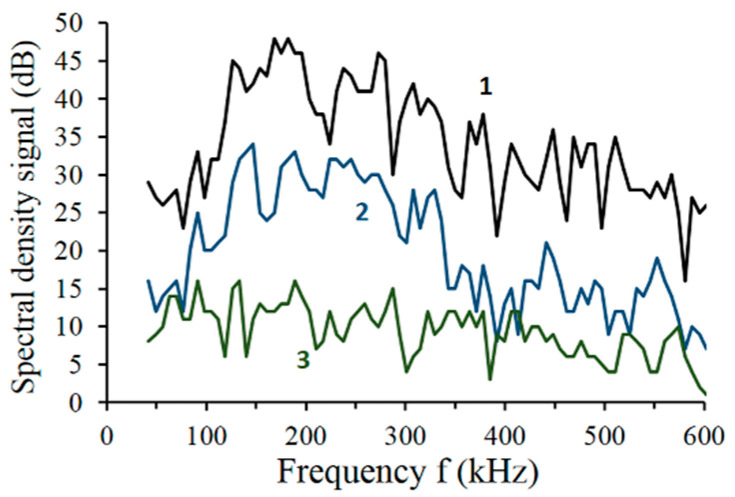
Spectral characteristics for bearing steel indicating energies of event patterns used in artificial neural networks (ANNs): 1—high energy AE events, 2—medium energy AE events, and 3—low energy AE events of background.

The formation of spectral characteristics, i.e., spectral density functions, enabled the comparison of the registered signal power in the selected frequency bands. The received spectral characteristics are shown in Figure 10.

The comparison of spectral characteristics allows a preliminary analysis of signal patterns adopted for the research. Spectral characteristics of high-energy events indicates that these events have local maxima within the frequency range, i.e., 100–300 kHz, while the value of the signal spectral density is within 30–45 dB. The AE events of low energy and low amplitude are similar to the acoustic noise background.

**Figure 11:**
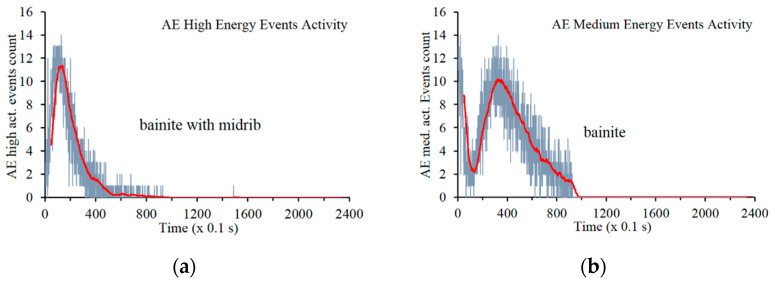
Relationship of the detected AE events correlated with patterns: (**a**) for high-energy AE events and (**b**) for medium-energy AE events.

The next stage of the research was to carry out a process of teaching the network. In this process, three pairs of patterns were applied at high, medium, and low AE signal power. A graph of the frequency of AE event occurrence was made as a function of time. The results of this relationship are illustrated in Figure 11.

The results obtained for the dependence of AE event frequency for high-energy patterns indicate the generation of a maximum number of events in the process, within approximately ten seconds after its start. The obtained results indicate the presence of bainite on midrib. The maximum intensity of AE events of medium energy occurs after 30 s of the entire process; after approx. 100 s, the phase transformation occurs farther, locally in a form of isolated incidents of acoustic emissions. The obtained results indicate the detection of bainitic transformation. The kinetics of this phenomenon indicates that midribs are the first elements that cause the occurrence of AE events. Low-energy AE events that are near noise background were neglected in this analysis. The cooling rate favors midrib nucleation, which significantly influences acceleration of the transformation. Mechanical stresses are an additional factor.

**Figure 12:**
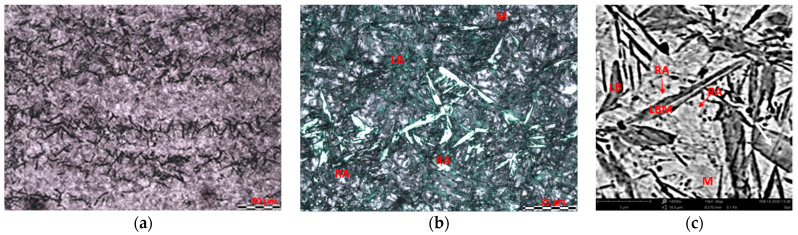
Microstructure of steel 100CrMnSi6-4 after austempering at 150 °C: (**a**) segregation of the microstructure; (**b**) observation of the microstructure details showing lower bainite plates (LB), retained austenite (RA), and martenzite (M) etched with Nital; and (**c**) a SEM micrograph of the lower bainite with midrib (LBM) formed at 150 °C.

It was observed on the microstructural (Figure 12) image that, while austempering the bearing steel at 150 °C, there were structural objects of the morphology of bainite with the midrib (Figure 12c), bainite, athermal martensite, and residual austenite (Figure 12b). A segregation of microstructures present in the image resulted from the carbide streaks along the rolling direction (Figure 12a). The streaks concern areas more enriched in carbon and alloyed elements, including carbon and chromium. In the first stage of the transformation, midribs dominate. The plates of midribs are twinned thin-plate martensite, and they are the first element being formed on the plate of bainite. The morphology of lower bainite with midrib is characterized by the layout of plates in the shape of a butterfly.

## 4. Discussion

A formation mechanism of the bainite structure has not been explained to the end. There are two schools suggesting different ways of explaining it. The first claims that ferrite, which is a main component of bainite, is generated as a result of a mechanism similar to the formation of Widmanstätten ferrite throughout the whole range of bainitic transformations. The representatives of this school explain the kinetics of bainitic transformation and the composition of individual phases by the effect of alloy components on the interface. According to this interpretation, the bainite carbides are generated inside, mainly on the ferrite/austenite interphase boundary [23]. In turn, the representatives of a different and dominant school of thinking believe that bainite can be described as an invariant-plane strain (IPS) with a large shear components [2]. It is concluded that the austenite/ferrite interface is of the same nature as martenitic transformation. Ferritic, supersaturated with carbon bainite components, stays in lower bainite, decarbonized due to the release of carbides inside the ferrite. The shearing mechanism in the phase transformation determines the morphology of the bainite plates formed. The transformation usually begins on the surface of the boundary of austenite grains due to the process of nucleation and growth of bainite plates. H. K. D. H. Bhadeshia developed a model of such a transformation mechanism [24]. The growth of bainitic ferrite is determined by a shearing mechanism and subplates grow without diffusion, whereas a certain part of the carbon diffuses to residual austenite in the moment of its nucleation [24,25]. In consequence, the growth of subsequent plates is inhibited due to the fact that austenite is more stable. The growth of further subplates is also stopped as a result of the resulting dislocations. These two essential processes should be considered in the formation of the bainitic structure. An independent nucleation of the subplate on surfaces of austenite grains and a nucleation due to autocatalysis on the formed subplates, which also takes place on lower bainite with a midrib, have been shown in this research. H. K. D. H. Bhadeshia [26,27] believes that the process of bainitic ferrite nucleation is similar to the nucleation of martensite. Therefore, according to the theory of martensite nucleation, nucleation is proportional to the driving force of nucleation [28].

The experiment results obtained by the authors of this research are consistent with the assumptions of predecessors concerning the occurrence of bainite below M_S_. Bearing steel was used in the presented research. Dilatometric tests (DMT) that confirm earlier observations of various authors were made to demonstrate the ability to generate bainite below M_S_. The tests were carried out with different cooling rates. Such conditions enabled elimination of the impact of stress and midribs that cause acceleration of bainitic transformations. In addition, to confirm the DMT results, the tests were performed using a highly sensitive research method that is acoustic emission (AE). Finally, the results of AE event frequency as a function of time were obtained. These results indicate the detection of signals from different physical events that have different event energies. Therefore, high-energy events indicate the occurrence of bainitic transformation initiated by midribs while events of medium energy indicate the presence of further bainitic transformation. Alloying agents such as chromium or manganese affect the value of the M_S_ temperature, plastic properties, or hardenability of steels, and thus, they also affect the shape of AE signals and the number of AE events. As a result, it significantly affects phase transformations while austempering. The phase transformation at the beginning of the process of isothermal hardening for steel used in this research is more intense and rapid in nature compared to bainitic transformation. Bainitic transformation is slower and, above all, has a wider range of occurrences. Bainitic transformation, after a specified time, occurs locally with single incidents of acoustic emission. The kinetics for high-energy events indicates that midribs are the first elements forming in this range. Midrib is a twinned thin-plated martensite. As demonstrated above, the formation of midribs occurs during the first seconds of the transformation. Midribs are the first elements on which bainite plates nucleate [11,12]. Moreover, on the midrib plane, a further mechanism of shearing bainite takes place. Bainitic transformation accelerates with lower temperature values. S. V. Radcliffe and E. C. Rollason [29] studied steels 0.65 ÷ 1.2 C wt% under isothermal conditions using the measurement method of electrical resistance. They found the “swing back” effect in the vicinity of M_S_ temperature, a phenomenon that is characterized by a clear acceleration of austenite decomposition. H. Okamoto and M. Oka [11,12] also confirmed that the acceleration of transformation initiation occurs near a M_S_ temperature of up to approx. 25 vol. % of bainite. However, these effects were interpreted by the influence of midribs on bainitic transformation.

Sources of acoustic emission of steel as a result of hardening are phase transformations, displacements, formation of twins, and microcracks [30]. According to A. Pawełek, the emission source may come from the collective features of the movements of dislocation groups including annihilation of the dislocations both inside and on the surface of the treated material. Therefore, there is a theoretical and experimental premises indicating that the effects of annihilation may have more significant impact on the acoustic effects compared with the acceleration of dislocations. The analysis of recorded AE signals allows you to determine the time of the occurrence of the effect that characterizes specific processes. A high resolution of the results obtained makes it easier to distinguish different events occurring during austempering. The programs applied here and the analytical method of AE signals enable the presentation in a graphical form of the differences between the effect that characterizes an incidental event and the event that occurred as a result of the development of phase transformations. An important element affecting the ability to record the received AE signal is also the geometry of the analyzed specimen. Changes in the dimensions of the tested specimen also significantly affect the development of transformations that take place inside the specimen.

## 5. Conclusions

In this study, it has been shown that the application of acoustic emission (AE) and neural network (NN) to phase transformation research in steels during isothermal quenching is a new and promising testing technique. In combination with dilatometry and microscopy, they may constitute a complementary set of research tools for the analysis of occurring phenomena.

1. As a result of tests made near the M_S_ temperature while austempering, on the basis of the results obtained in a form of the graphs of the dilatometric measurement, and based on AE signals as well as on microstructure images, it is concluded that the product obtained from phase transformation was bainite.
2. The research on acoustic emission illustrates natural conditions associated with cooling (quenching). Midribs and stresses accelerate bainitic transformation. In view of the intense effect of midribs and mechanic stresses on the kinetics of bainitic transformation, in dilatometric tests, the quenching rate decreased.
3. Acoustic emission belongs to a group of nondestructive tests, the aim of which is to identify processes and transformations on the macro and micro scales. Different events of acoustic emission were found in the recorded signals.
4. The AE signals are generated due to the release of energy accumulated inside the material. The sources of these signals are phase transformations, a shear transformation, and dislocation events. Advanced computer programs enable you to visualize the recorded AE signals. The analyzed signals can be represented as spectral characteristics in the form of spectrograms.
5. An analysis of the results of AE measurements recorded during austempering at 150 °C showed that the essential acoustical effects occurred in particular in the first 100 s of a registered AE signal. The tracking trials of transformation kinetics allowed the conclusion that, in the first 40 s, there is bainitic transformation with a midrib, whereas, after that time, further bainitic transformation of lower energy is started.
6. More and more companies, especially from the automotive, aviation, and machine industries, are investing in hardening plants. Therefore, new solutions and technologies in this field are still being searched for. Any attempts to solve material problems and to consolidate knowledge about physical phenomena can facilitate faster and more effective work and can help optimize production processes.
7. The research methods used in this work enable the analysis and identification of phase changes. Knowledge of these phenomena can consequently aid the design of heat treatment operations and, at the same time, allow the shaping of heat-treated elements without side effects in the form of deformations, cracks, and delamination.

## Figures and Tables

**Table 1:** Chemical composition of bearing steel 100CrMnSi6-4 (wt%).

C	Mn	Si	P	S	Cr	Ni	Mo	Al	Cu
0.98	1.10	0.6	0.012	0.009	1.51	0.08	0.02	0.018	0.20

## Data Availability

Not applicable.
